# Preemptive Immunotherapy for Minimal Residual Disease in Patients With t(8;21) Acute Myeloid Leukemia After Allogeneic Hematopoietic Stem Cell Transplantation

**DOI:** 10.3389/fonc.2021.773394

**Published:** 2022-01-06

**Authors:** Shuang Fan, Meng-Zhu Shen, Xiao-Hui Zhang, Lan-Ping Xu, Yu Wang, Chen-Hua Yan, Huan Chen, Yu-Hong Chen, Wei Han, Feng-Rong Wang, Jing-Zhi Wang, Xiao-Su Zhao, Ya-Zhen Qin, Ying-Jun Chang, Kai-Yan Liu, Xiao-Jun Huang, Xiao-Dong Mo

**Affiliations:** ^1^ Peking University People’s Hospital, Peking University Institute of Hematology, National Clinical Research Center for Hematologic Disease, Beijing Key Laboratory of Hematopoietic Stem Cell Transplantation, Beijing, China; ^2^ Peking-Tsinghua Center for Life Sciences, Academy for Advanced Interdisciplinary Studies, Peking University, Beijing, China; ^3^ Research Unit of Key Technique for Diagnosis and Treatments of Hematologic Malignancies, Chinese Academy of Medical Sciences, Beijing, China

**Keywords:** *RUNX1-RUNX1T1*, allogeneic hematopoietic stem cell transplantation, preemptive, interferon, donor lymphocyte infusion

## Abstract

In patients with t(8;21) acute myeloid leukemia (AML), recurrent minimal residual disease (MRD) measured by *RUNX1-RUNX1T1* transcript levels can predict relapse after allogeneic hematopoietic stem cell transplantation (allo-HSCT). This study aimed to compare the efficacy of preemptive interferon (IFN)-α therapy and donor lymphocyte infusion (DLI) in patients with t(8;21) AML following allo-HSCT. We also evaluated the appropriate method for patients with different levels of *RUNX1-RUNX1T1* transcripts. In this retrospective study, consecutive patients who had high-risk t(8;21) AML and received allo-HSCT were enrolled. The inclusion criteria were as follows: (1) age ≤65 years; (2) regained MRD positive following allo-HSCT. MRD positive was defined as the loss of a ≥4.5-log reduction and/or <4.5-log reduction in the *RUNX1-RUNX1T1* transcripts, and high-level, intermediate-level, and low-level MRDs were, respectively, defined as <2.5-log, 2.5−3.5-log, and 3.5−4.5-log reductions in the transcripts compared with the pretreatment baseline level. Patients with positive *RUNX1-RUNX1T1* could receive preemptive IFN-α therapy or DLI, which was primarily based on donor availability and the intentions of physicians and patients. The patients received recombinant human IFN-α-2b therapy by subcutaneous injection twice a week every 4 weeks. IFN-α therapy was scheduled for six cycles or until the *RUNX1-RUNX1T1* transcripts were negative for at least two consecutive tests. The rates of MRD turning negative for patients with low-level, intermediate-level, and high-level *RUNX1-RUNX1T1* receiving IFN-α were 87.5%, 58.1%, and 22.2%, respectively; meanwhile, for patients with intermediate-level and high-level *RUNX1-RUNX1T1* receiving DLI, the rates were 50.0% and 14.3%, respectively. For patients with low-level and intermediate-level *RUNX1-RUNX1T1*, the probability of overall survival at 2 years was higher in the IFN-α group than in the DLI group (87.6% *vs.* 55.6%; *p* = 0.003). For patients with high levels of *RUNX1-RUNX1T1*, the probability of overall survival was comparable between the IFN-α and DLI groups (53.3% *vs.* 83.3%; *p* = 0.780). Therefore, patients with low-level and intermediate-level *RUNX1-RUNX1T1* could benefit more from preemptive IFN-α therapy compared with DLI. Clinical outcomes were comparable between preemptive IFN-α therapy and DLI in patients with high-level *RUNX1-RUNX1T1*; however, they should be further improved.

## 1 Introduction

Acute myeloid leukemia (AML) with t(8;21) is a heterogeneous disease, and relapse can occur in 40–50% of patients treated with chemotherapy alone, even if it is considered to have a good prognosis (1, 2). Minimal residual disease (MRD) after chemotherapy can predict the relapse of t(8;21) AML (3–6), and allogeneic hematopoietic stem cell transplantation (allo-HSCT) can further decrease relapse and improve survival in patients with persistent *RUNX1-RUNX1T1* after chemotherapy (5, 7–9). However, relapse remains experienced by nearly 20% of patients following allo-HSCT (10).

Regular monitoring of MRD after allo-HSCT can identify patients with a higher risk of relapse (11, 12). The MRD measured by the level of *RUNX1-RUNX1T1* transcript has been identified as an effective predictor of relapse in patients with t(8;21) AML after allo-HSCT (13, 14). Therefore, intervention directed by MRD (i.e., preemptive intervention) is a rational option for relapse prophylaxis. One of the most critical immunotherapies after allo-HSCT is donor lymphocyte infusion (DLI) (15–17). Wang et al. (14) reported that preemptive DLI could prevent relapse and improve survival in patients with t(8;21) AML. Interferon-α (IFN-α) is another important immunotherapy after allo-HSCT (18–22); Mo et al. (20) reported that the survival of patients with MRD positive without any intervention was significantly lower than those receiving preemptive IFN-α therapy (20). Therefore, IFN-α therapy and DLI could improve the prognosis of patients with MRD following allo-HSCT. However, which preemptive intervention is more superior for t (8;21) AML patients receiving allo-HSCT is still unclear. Mo et al. (21) reported that the prognosis of preemptive DLI and IFN-α therapy was comparable, but their study included a small sample size of patients with t(8;21) AML. To date, no studies have compared the efficacy of preemptive DLI and IFN-α therapy in patients with t(8;21) AML.

Furthermore, we observed that *RUNX1-RUNX1T1* transcript levels influenced the efficacy of preemptive IFN-α therapy; however, the influence of MRD levels on IFN-α therapy could not be further evaluated due to a small sample size of patients with higher levels of *RUNX1-RUNX1T1* transcripts (20). In contrast, Wang et al. (14) reported that patients with a higher level of *RUNX1-RUNX1T1* transcript could still benefit from DLI. Therefore, patients with different levels of *RUNX1-RUNX1T1* transcript may benefit from different interventions. However, no studies have compared the efficacy of preemptive DLI and IFN-α therapy at different levels of *RUNX1-RUNX1T1* transcript and the selection of appropriate preemptive interventions according to *RUNX1-RUNX1T1* transcript levels remains unknown.

Therefore, this retrospective study aimed to compare the efficacy of preemptive DLI and IFN-α therapy in patients with t(8;21) AML following allo-HSCT. Furthermore, we also evaluated the appropriate intervention methods for patients with different levels of *RUNX1-RUNX1T1* transcripts.

## 2 Methods

### 2.1 Patients

Consecutive patients who had high-risk t(8;21) AML and received allo-HSCT at the Peking University Institute of Hematology (PUIH) were enrolled. The inclusion criteria were as follows: (1) were ≤65 years old (2) and regained MRD positive following allo-HSCT (5).

The exclusion criteria for IFN-α therapy were as follows: (1) active graft-versus-host disease (GVHD); (2) active and uncontrolled infections; (3) severe myelosuppression; (4) organ failure; and (5) hematologic relapse.

The exclusion criteria for DLI were as follows: (1) active GVHD; (2) active and uncontrolled infections; (3) organ failure; and (4) hematologic relapse (20).

One hundred and four patients were enrolled between October 1, 2013 and February 28, 2021 (**Table 1**). Forty-two patients were previously reported by Mo et al. (23), and in this study, they were followed up further. The endpoint analysis of the last follow-up was on September 1, 2021.

**Table 1:** Patient characteristics.

Characteristics	IFN-α group (*n* = 88)	DLI group (*n* = 16)	*p*-value
Median age at allo-HSCT (years (range))	27.5 (7–57)	30.5 (4–49)	0.836
Median time from allo-HSCT to interventions (days (range))	139 (36–710)	103.5 (46–217)	0.522
First CR induction courses (*n* (%))			
1	68 (77.3)	8 (50.0)	0.024
>1	20 (22.7)	8 (50.0)	
Additional chromosomal abnormality			
No	78 (88.7)	14 (87.5)	0.896
Yes	10 (11.3)	2 (12.5)	
*c-KIT* gene at diagnosis (*n* (%))			
Mutation	39 (44.3)	10 (62.5)	0.182
Wild type	49 (55.7)	6 (37.5)	
Sex (*n* (%))			
Male	52 (59.0)	9 (56.3)	0.833
Female	36 (40.9)	7 (43.8)	
Disease status at allo-HSCT (*n* (%))			
CR1	72 (81.8)	10 (62.5)	0.083
>CR1	16 (18.2)	6 (37.5)	
RUNX1-RUNX1T1 transcript levels before HSCT (*n* (%))			
3.5-4.5-log reduction	13 (14.8)	1 (6.3)	0.027
2.5-3.5-log reduction	42 (47.7)	4 (25.0)	
<2.5-log reduction	33 (37.5)	11 (68.7)	
Donor-recipient sex match (*n* (%))			
Male-male	33 (37.5)	6 (37.5)	0.527
Male-female	27 (30.7)	4 (25)	
Female-male	18 (20.5)	3 (18.8)	
Female-female	10 (11.4)	3 (18.8)	
Donor type (*n* (%))			
HLA-identical sibling donor	20 (22.7)	4 (25.0)	0.869
HLA-haploidentical related donor	65 (73.9)	12 (75.0)	
HLA-unrelated donor	3 (3.4)	0 (0.0)	
Number of HLA-A, HLA-B, HLA-DR mismatches (*n* (%))			
0	23 (26.1)	4 (25.0)	0.590
1	3 (3.4)	0 (0.0)	
2	15 (17.0)	2 (12.5)	
3	47 (53.4)	10 (62.5)	
RUNX1-RUNX1T1 level before interventions (*n* (%))^a^			
Low	48 (54.5)	3 (18.8)	0.001
Intermediate	31 (35.2)	6 (37.5)	
High	9 (10.2)	7 (43.8)	
Median duration of immunosuppressive therapy before MRD occurred (days (range))	72 (21–324)	47.5 (21–199)	0.476
Discontinuing immunosuppressions before interventions (*n* (%))	49 (55.7)	8 (50.0)	0.676
aGVHD before MRD positive (*n* (%))	31 (35.2)	8 (50.0)	0.264
cGVHD before MRD positive (*n* (%))	3 (3.4)	0 (0)	0.456

allo-HSCT, allogeneic hematopoietic stem cell transplantation; CR, complete remission; DLI, donor lymphocyte infusion; GVHD, graft-versus-host disease; HLA, human leukocyte antigen; IFN-α, interferon-α.

a High-level, intermediate-level, and low-level MRDs were respectively defined as <2.5-log, 2.5- to 3.5-log, and 3.5- to 4.5-log reductions in the RUNX1-RUNX1T1 transcripts when compared with the pretreatment baseline level.

### 2.2 Transplant Regimens

Cytosine arabinoside, busulfan, cyclophosphamide (CY), and simustine were included in the preconditioning. The human leukocyte antigen (HLA)-unrelated donor (URD) and HLA-haploidentical donor (HID) groups received rabbit antithymocyte globulin (ATG, **Supplementary Methods**) (24–26). HID HSCT recipients received ATG and low-dose posttransplant CY (PTCY) for GVHD prophylaxis according to the protocol registered at http://clinicaltrials.gov/NCT02412423 (**Supplementary Methods** and **Supplementary Figure S1**) (24–28). Protocols for stem cell harvesting, donor selection, and HLA typing have been previously described in detail (29–32).

### 2.3 MRD Monitoring and Definition

The protocol for *RUNX1-RUNX1T1* monitoring after allo-HSCT was performed according to the protocol of PUIH (5, 13). The definition of MRD positive was a loss of *RUNX1-RUNX1T1* transcripts ≥4.5-log reduction and/or the <4.5-log reduction (20. Low-level, intermediate-level, and high-level MRD was defined as a reduction in transcripts of 3.5−4.5-log, 2.5−3.5-log, and <2.5-log, respectively, compared with the baseline level before treatment.

### 2.4 Protocol for Preemptive DLI and IFN-α Therapy

In this retrospective study, patients with positive *RUNX1-RUNX1T1* received preemptive IFN-α therapy or DLI before hematologic relapse after allo-HSCT (12). The therapeutic option was primarily based on donor availability and the intentions of physicians and patients.

The patients received recombinant human IFN-α-2b therapy by subcutaneous injection twice a week every 4 weeks. IFN-α therapy was scheduled for six cycles or until the *RUNX1-RUNX1T1* transcripts were negative for at least two consecutive tests (**Supplementary Methods**). IFN-α therapy could be prolonged upon the request of patients. IFN-α therapy was discontinued in patients with grade ≥3 toxicity, severe infection, severe GVHD, nonrelapse mortality (NRM), or relapse.

Granulocyte colony-stimulating factor (G-CSF)-mobilized peripheral blood stem cells were administered instead of unstimulated donor blood lymphocytes. All patients received short-term immunosuppressive drugs after DLI. Patients could receive chemotherapy 48–72 h before DLI (i.e., chemo-DLI) (**Supplementary Methods**) (16, 17).

MRD status was regularly monitored at 1, 2, 3, 4.5, 6, 9, and 12 months after preemptive intervention and at 6-month intervals thereafter.

**Figure 1:**
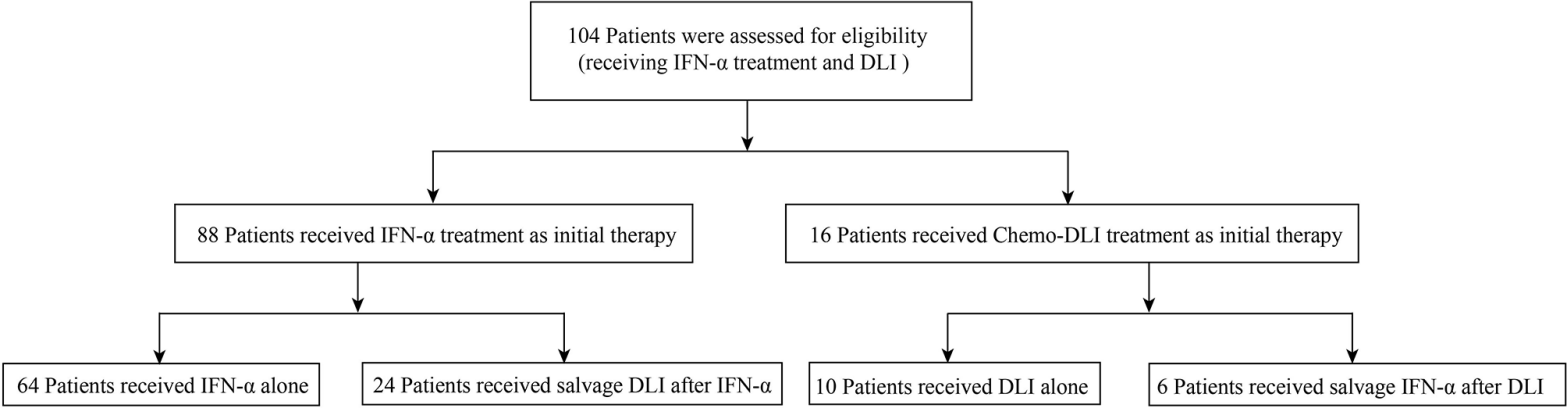
Diagram of patients enrolled.

For patients with persistent and increasing levels of MRD (e.g., levels of *RUNX1-RUNX1T1* transcripts increased by 1-log) or those who regained MRD positive after receiving MRD-negative status, if they were in the IFN-α group, they could be switched to the DLI group and vice versa (**Figure 1**).

### 2.5 Diagnosis and Therapy of GVHD After Preemptive Immunotherapy

GVHD diagnosis and therapy were based on common international criteria (33–38).

### 2.6 Definition and Assessment

Relapse was defined according to common international criteria (39). Patients who showed relapse were not considered to have MRD. NRM was defined as death without relapse or disease progression. Leukemia-free survival (LFS) was defined as a lifetime with continuous complete remission (CR). The event of overall survival (OS) was the death of any cause.

### 2.7 Statistical Analysis

The *χ* ^2^ and Fisher’s exact tests were used to compare categorical variables. The Mann–Whitney *U* test was used to compare continuous variables. The cumulative incidences of relapse, NRM, and GVHD were calculated using competing risk analyses (40). The probabilities of OS and LFS were estimated using the Kaplan−Meier method. The full analysis set (FAS) included all participants who received DLI (*n* = 16) or IFN-α (*n* = 88) as initial therapy at the time of MRD positive. The per-protocol set (PPS) analysis included patients who received DLI (*n* = 10) or IFN-α therapy (*n* = 64) alone and those who received both DLI and IFN-α (*n* = 30) were excluded from the PPS analysis.

Cox proportional hazards regression with a backward stepwise model selection approach was used to estimate hazard ratios for clinical outcomes in a multivariate analysis. The following variables were included: sex, disease status (>CR1 *vs.* CR1), c-KIT gene at diagnosis (wild type *vs.* mutation), pre-HSCT *RUNX1-RUNX1T1* level (high-level *vs.* intermediate-level *vs.* low-level), donor type (identical sibling donor *vs.* alternative donor), and *RUNX1-RUNX1T1* level before preemptive interventions (high-level *vs.* intermediate-level *vs.* low-level). Independent variables with *p* < 0.05 were identified as statistically significant, and *p* > 0.1 was sequentially excluded from the model. Data analyses were performed primarily using SPSS software (SPSS Inc., Chicago, IL, USA) and the R software package (version 4.1.1; http://www.r-project.org).

## 3 Results

### 3.1 Patient Characteristics

**Figure 2:**
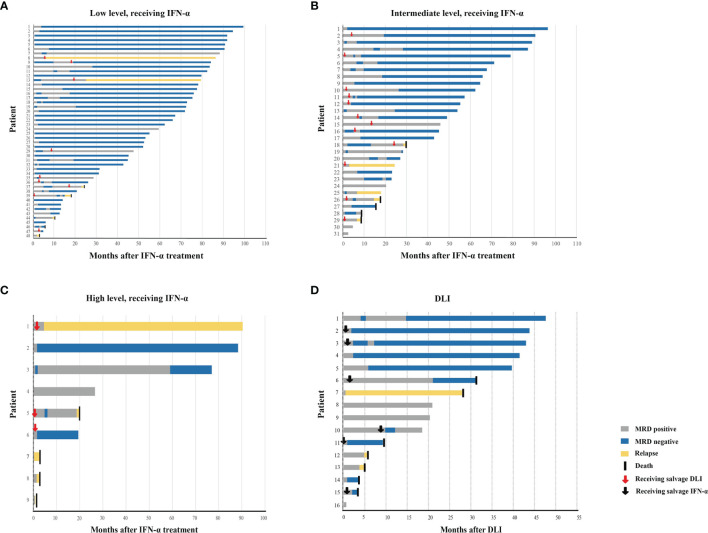
Response. Swimmer plot displayed patients receiving preemptive IFN-α therapy with low-level *RUNX1-RUNX1T1* **(A)**, intermediate-level *RUNX1-RUNX1T1* **(B)**, and high-level *RUNX1-RUNX1T1* transcripts **(C)**, respectively, and patients receiving preemptive DLI **(D)**. IFN, interferon; DLI, donor lymphocyte infusion.

Patient characteristics are shown in **Table 1**, **Figure 2**, and **Supplemental Table S1**. In particular, six HID HSCT recipients received ATG and low-dose PTCY for GVHD prophylaxis. A total of 51, 37, and 16 patients showed low-level, intermediate-level, and high-level *RUNX1-RUNX1T1*, respectively, after allo-HSCT. We observed that donor type, *Kit* mutation, other karyotypic abnormalities, and duration of immunosuppressive therapy before MRD were not associated with posttransplant *RUNX1-RUNX1T1* levels (**Supplementary Tables S2**, **S3** and **Supplementary Figure S1**); however, pre-transplant transcripts were associated with post-transplant *RUNX1-RUNX1T1* levels (**Supplementary Table S2** and **Supplementary Figure S1C**).

Eighty-eight patients received IFN-α as initial therapy. The median number of cycles of IFN-α therapy was 3 cycles (range, 1–26 cycles), and 24 of them received salvage DLI (chemo-DLI, 20; DLI alone, 4) after IFN-α therapy. Sixteen patients received DLI as initial therapy (chemo-DLI, 10; DLI alone, 6), and six of them received IFN-α as salvage therapy after DLI. The causes of NRM are infection, diffused alveolar hemorrhage, and GVHD (**Supplementary Table S4**). The cumulative incidences of relapse, NRM, LFS, and OS at 2 years after preemptive interventions were 16.8% [95% confidence interval (CI), 8.7%−24.8%] versus 19.6% (95% CI, 0.0%−40.5%) (*p* = 0.810), 3.6% (95% CI, 0.0%−7.7%) versus 20.1% (95% CI, 0.0%−41.3%) (*p* = 0.001), 78.2% (95% CI, 69.8%−87.7%) versus 60.3% (95% CI, 40.0%−90.9%) (*p* = 0.023), and 84.2% (95% CI, 76.6%−92.5%) versus 66.7% (95% CI, 46.6%−95.3%) (*p* = 0.004), respectively, for the IFN-α and DLI groups.

### 3.2 Correlation Between *RUNX1-RUNX1T1* Status Following Allo-HSCT and Preemptive Interventions

#### 3.2.1 Low-Level *RUNX1-RUNX1T1* Before Immunotherapy

Of the 48 patients who received IFN-α as initial therapy, 42 of them achieved MRD negative (87.5%, **Supplementary Table S5**), and the median duration from intervention to MRD turning negative was 43.5 days (range, 11–846 days). Nine patients received salvage DLI (chemo-DLI, 8; DLI alone, 1) after IFN-α therapy (regained positive after achieving negative, 5; persistent positive, 4), and three patients receiving chemo-DLI (3/8, 37.5%) achieved MRD negative.

Three patients received DLI as initial therapy (chemo-DLI, 1; DLI alone, 2), but none of them achieved MRD negative (**Supplementary Table S5**). A patient received salvage IFN-α after chemo-DLI and achieved MRD negative thereafter.

#### 3.2.2 Intermediate-Level *RUNX1-RUNX1T1* Before Immunotherapy

Of the 31 patients who received IFN-α treatment as initial therapy, 18 of them achieved MRD negative (58.1%, **Supplementary Table S5**), and the median duration from intervention to MRD turning negative was 117 days (range, 16–556 days). Twelve patients received salvage DLI (chemo-DLI, 9; DLI alone, 3) after IFN-α therapy (regained positive after achieving negative, 2; persistent positive, 10), and seven of them (58.3%) achieved MRD negative (chemo-DLI, 6; DLI alone, 1).

Six patients received DLI as initial therapy (chemo-DLI, 2; DLI alone, 4), three of them (50.0%) achieved MRD negative (**Supplementary Table S5**), and the duration from intervention to MRD turning negative was 25, 121, and 174 days, respectively. Three patients with persistent MRD positive received salvage IFN-α therapy after DLI, and all of them achieved MRD negative thereafter.

#### 3.2.3 High-Level *RUNX1-RUNX1T1* Before Immunotherapy

Nine patients received IFN-α therapy as initial therapy; two achieved MRD negative (22.2%, **Supplementary Table S5**), and the duration from intervention to MRD turning negative was 23 and 48 days, respectively. Three patients with persistent MRD positive received salvage chemo-DLI after IFN-α therapy. Although two of them (66.7%) achieved a transient MRD negative after that, both experienced relapse.

Seven patients received chemo-DLI as initial therapy, 1 (14.3%) achieved MRD negative (**Supplementary Table S5**), and the duration from intervention to MRD turning negative was 68 days. Two patients with persistent MRD positive received salvage IFN-α therapy after chemo-DLI, and both achieved MRD-negative status afterward.

### 3.3 Chronic GVHD After Preemptive Immunotherapy

The cumulative incidence of total chronic GVHD (cGVHD) at 2 years after preemptive immunotherapy was 45.1% (95% CI, 32.4%−57.8%) in patients receiving IFN-α therapy alone, 57.1% (95% CI, 4.4%−100.0%) in patients receiving DLI alone, and 75.3% (95% CI, 58.6%−92.0%) in patients receiving both DLI and IFN-α therapy (*p* = 0.154). The cumulative incidence of severe cGVHD at 2 years after preemptive immunotherapy was 3.2% (95% CI, 0.0%−7.5%) in patients receiving IFN-α therapy alone, 0.0% in patients receiving DLI alone, and 10.1% (95% CI, 0.0%−21.2%) in patients receiving both DLI and IFN-α therapy (*p* = 0.288)

### 3.4 Relapse, NRM, and Survival After Preemptive Immunotherapy

#### 3.4.1 FAS

**Figure 3:**
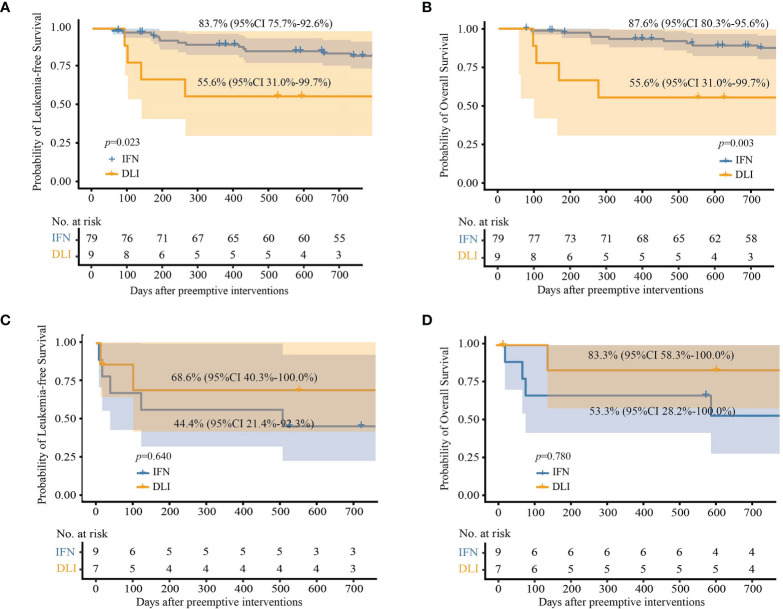
Probabilities of survival at 2 years after preemptive immunotherapies in full analysis set. **(A)** Leukemia-free survival in patients with low- and intermediate-level *RUNX1-RUNX1T1*; **(B)** overall survival in patients with low- and intermediate-level *RUNX1-RUNX1T1*; **(C)** leukemia-free survival in patients with high-level *RUNX1-RUNX1T1*; **(D)** overall survival in patients with high-level *RUNX1-RUNX1T1*.

For patients with low-level and intermediate-level *RUNX1-RUNX1T1* (i.e., 2.5−4.5-log reduction), the 2-year cumulative incidence of relapse after the intervention was comparable between the IFN-α and DLI groups, but the IFN-α group showed a lower cumulative incidence of NRM (**Table 2**). The probability of survival at 2 years in the IFN-α group was also significantly better than that of the DLI group (**Table 2** and **Figures 3A, B**). Particularly, for patients with low levels of *RUNX1-RUNX1T1*, the cumulative incidence of relapse, NRM, LFS, and OS at 2 years after IFN-α therapy was 11.3% (95% CI, 1.8%−20.7%; 2.1% (95% CI, 0.0%−6.3%), 86.6% (95% CI, 77.1%−97.2%), and 88.8% (95% CI, 79.8%−98.6%), respectively.

**Table 2:** The 2-year cumulative incidence of relapse, NRM, LFS, and OS after preemptive interventions.

	IFN-α		DLI		*p*-value
	n	Cumulative incidence (95% CI)	n	Cumulative incidence (95%CI)	
**Low- and intermediate-level *RUNX1-RUNX1T1* ^a^**					
Full analysis set	79		9		
Relapse	11	12.2% (4.7%–19.8%)	1	11.1% (0%–33.3%)	0.870
NRM	3	4.1% (0%–8.6%)	3	33.3% (0.1–66.5%)	0.001
LFS	65	83.7% (75.7%–92.6%)	5	55.6% (31.0%–99.7%)	0.023
OS	69	87.6% (80.3%–95.6%)	5	55.6% (31.0%–99.7%)	0.003
Per protocol set	58		5		
Relapse	3	5.4% (0.0%–11.3%)	1	20.0% (0.0%–60.4%)	0.203
NRM	3	5.6% (0.0%–11.8%)	1	20.0% (0.0%–59.2%)	0.202
LFS	52	89.1% (81.1%–97.7%)	3	60.0% (29.3%–100.0%)	0.030
OS	53	90.8% (83.5%–98.8%)	3	60.0% (29.3%–100.0%)	0.017
**High-level *RUNX1-RUNX1T1* ^a^**					
Full analysis set	9		7		
Relapse	5	55.6% (20.0%–91.1%)	2	31.4% (0.0%–71.8%)	0.422
NRM	0	0.0%	1	0.0%	0.326
LFS	4	44.4% (21.4%–92.3%)	4	68.6% (40.3%–100.0%)	0.640
OS	5	53.3% (28.2%–100.0%)	4	83.3% (58.3%–100.0%)	0.780
Per protocol set	6		5		
Relapse	3	50.0% (4.7%–95.3%)	2	46.7% (0.0%–100.0%)	0.767
NRM	0	0.0%	0	0.0%	
LFS	3	50.0% (22.5%–100.0%)	3	53.3% (21.4%–100.0%)	0.770
OS	3	50.0% (22.5%–100.0%)	3	75.0% (42.6%–100.0%)	0.710

CI, confidence interval; DLI, donor lymphocyte infusion; IFN-α, interferon-α; LFS, leukemia-free survival; NRM, non-relapse mortality; OS, overall survival.

^a^High-level, intermediate-level, and low-level MRDs were respectively defined as <2.5-log, 2.5- to 3.5-log, and 3.5- to 4.5-log reductions in the RUNX1-RUNX1T1 transcripts when compared with the pretreatment baseline level.

The full analysis set included all participants who received IFN-α or DLI as initial treatments at the time of MRD positive and those who received both IFN-α and DLI were included. The per-protocol set analysis included the patients who received IFN-α or DLI alone, and those who received both IFN-α and DLI were excluded.

For patients with high-level *RUNX1-RUNX1T1* (i.e., <2.5-log reduction), the probabilities of survival at 2 years after intervention were all comparable between the IFN-α and DLI groups (**Table 2** and **Figures 3C, D**).

In multivariate analysis, for patients receiving preemptive IFN-α therapy, the relapse and survival of the low-level and intermediate-level *RUNX1-RUNX1T1* groups were superior to those of the high-level group (**Table 3**). In addition, identical sibling donors also predicted a high risk of relapse and poorer survival.

**Table 3:** Multivariate analysis of risk factors for the 2-year clinical outcomes after preemptive IFN-α therapy in full analysis set.

Outcome	HR (95% CI)	*p*-value
**Relapse**		
Disease status prior to allo-HSCT		
CR1	1	
>CR1	3.02 (1.07–8.48)	0.036
MRD level before IFN-α therapy^a^		
High-level	1	
Intermediate-level	0.18 (0.05–0.65)	0.009
Low-level	0.16 (0.05–0.53)	0.003
Donor type		
Alternative donor	1	
HLA-identical donor	6.04 (2.18–16.72)	0.001
**Treatment failure as defined by OS**		
MRD level before IFN-α therapy^a^		
High-level	1	
Intermediate-level	0.23 (0.06–0.89)	0.034
Low-level	0.18 (0.05–0.67)	0.011
Donor type		
Alternative donor	1	
HLA-identical donor	8.49 (2.77–26.01)	<0.001
**Treatment failure as defined by LFS**		
MRD level before IFN-α therapy^a^		
High-level	1	
Intermediate-level	0.25 (0.08-0.81)	0.021
Low-level	0.18 (0.06-0.59)	0.004
Donor type		
Alternative donor	1	
HLA-identical donor	6.09 (2.41-15.40)	<0.001

allo-HSCT, allogeneic hematopoietic stem cell transplantation; CI, confidence interval; HLA, human leukocyte antigen; HR, hazard ratio; IFN, interferon; LFS, leukemia-free survival; MRD, minimal residual disease; OS, overall survival.

^a^High-level, intermediate-level, and low-level MRDs were respectively defined as <2.5-log, 2.5- to 3.5-log, and 3.5- to 4.5-log reductions in the RUNX1-RUNX1T1 transcripts when compared with the pretreatment baseline level.

None of variables was significantly associated with increased NRM in multivariate analysis.

#### 3.4.2 PPS Analysis

In this analysis, patients who received both DLI and IFN-α treatment were excluded, and 64 and 10 patients in the IFN-α and DLI groups, respectively (**Table 2**).

**Figure 4:**
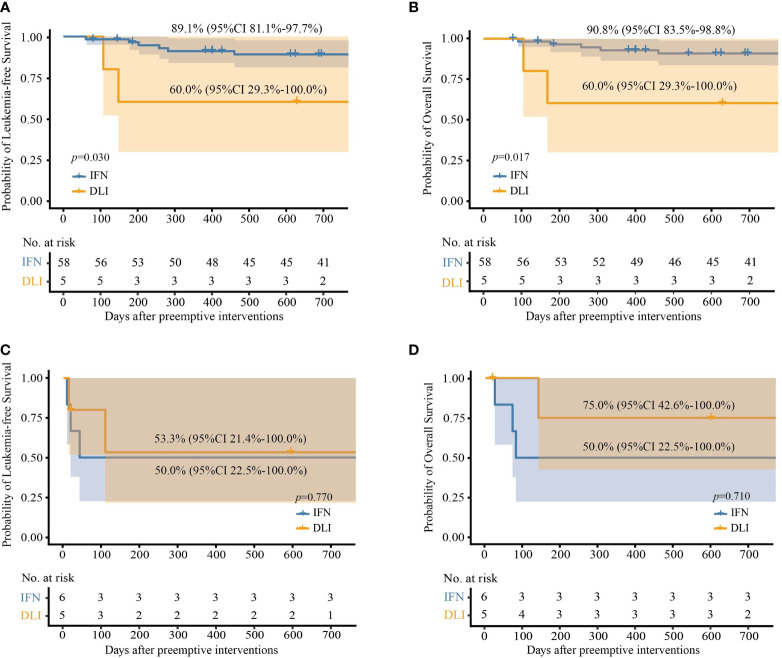
Probabilities of survival at 2 years after preemptive immunotherapies in per protocol set. **(A)** Leukemia-free survival in patients with low- and intermediate-level *RUNX1-RUNX1T1*; **(B)** overall survival in patients with low- and intermediate-level *RUNX1-RUNX1T1*; **(C)** leukemia-free survival in patients with high-level *RUNX1-RUNX1T1*; **(D)** overall survival in patients with high-level *RUNX1-RUNX1T1*.

For patients with low-level and intermediate-level *RUNX1-RUNX1T1*, the IFN-α group also showed significantly better OS and LFS rates than those of the DLI group (**Table 2** and **Figures 4A, B**). Particularly, for patients with low-level *RUNX1-RUNX1T1*, the incidence of NRM, relapse, LFS, and OS at 2 years after IFN-α therapy was 5.2% (95% CI, 0.0%−12.3%), 2.6% (95% CI, 0.0%−7.6%), 92.2% (95% CI, 84.2%−100.0%), and 92.2% (95% CI, 84.2%−100.0%), respectively.

For patients with high-level *RUNX1-RUNX1T1*, the probabilities of survival at 2 years after the intervention were all comparable between the IFN-α and DLI groups (**Table 2** and **Figures 4C, D**).

In multivariate analysis, for patients receiving preemptive IFN-α treatment, the OS of the low-level and intermediate-level *RUNX1-RUNX1T1* group was superior to that of the high-level group (**Supplementary Table S6**). Furthermore, identical sibling donors predicted poorer survival.

#### 3.4.3 Analysis of Patients Who Received Both IFN-α and Chemo-DLI

A total of 30 patients receiving both IFN-α and DLI were included in this analysis (DLI followed by IFN, *n* = 6; IFN followed by DLI, *n* = 24; **Figure 1**).

For patients with low-level and intermediate-level *RUNX1-RUNX1T1*, the cumulative incidence of NRM at 2 years was lower in IFN-α followed by DLI group (0% *vs.* 50.0%, 95% CI, 0.0%−100.0%; *p* =0.001) than those in DLI followed by the IFN-α group, but the probability of relapse and survival were all comparable between the groups (**Supplementary Table S7**).

Three patients with high-level *RUNX1-RUNX1T1* received salvage DLI after IFN-α therapy. Two of them achieved MRD negative, but one of them experienced relapse and died. The patient with persistent MRD positive also experienced a relapse. Two patients received salvage IFN-α after DLI, one of whom achieved MRD negative but died from pneumonia, and the other achieved MRD negative and persistent LFS until the last follow-up.

## 4 Discussion

This study showed that patients with low-level and intermediate-level *RUNX1-RUNX1T1* could benefit from preemptive IFN-α therapy. The clinical outcomes of preemptive IFN-α therapy and DLI in patients with high-level *RUNX1-RUNX1T1* were unsatisfactory. To our knowledge, this is the first study to compare the efficacy of preemptive IFN-α therapy and DLI in a population of patients with a specific disease [i.e., t(8;21) AML] following allo-HSCT.

This study showed that the NRM rate was <10% in patients who received preemptive IFN-α therapy, similar to our previous studies (20, 22). Klingemann et al. (41) also reported that no life-threatening complications occurred during IFN-α therapy after HSCT. And, the results showed that the rate of NRM appeared to be higher in the DLI group than in the IFN-α group for patients with low-level and intermediate-level *RUNX1-RUNX1T1*, suggesting that the safety of IFN-α therapy may be more satisfactory in these patients. This may be because IFN-α therapy was administered in divided doses and could be adjusted if early signs of toxicity or GVHD were observed.

The graft-versus-leukemia (GVL) effect, which is strongly associated with cGVHD (42, 43), is the main mechanism for IFN-α clearing of MRD (44). The incidence of cGVHD was comparable between the preemptive DLI and IFN-α therapy groups (51.1% *vs.* 62.5%, *p* = 0.405). Therefore, the capacity to induce GVL was comparable between IFN-α therapy and DLI (19, 21) and could contribute to the similar rate of MRD achieving negative results between these two methods. However, cGHVD, particularly severe cGVHD, can influence quality of life (45, 46) and cause mortality (47). In this study, only 3.2% of patients experienced severe cGVHD, and no patients died from GVHD after IFN-α therapy. Therefore, the intensity of IFN-α-induced cGVHD was easy to be controlled.

We observed that the relapse rate was nearly one-third (27.4%) even in patients with low-level *RUNX1-RUNX1T1* after transplantation (Qin et al., data unpublished) if no preemptive interventions were administered. In patients with low-level and intermediate-level *RUNX1-RUNX1T1*, most of them achieved MRD negative after IFN-α therapy. The rate of relapse was low, and the rate of survival was >80%, particularly for those with low-level *RUNX1-RUNX1T1*. In our previous study, the relapse and survival rates were 8% and 75%, respectively, for patients who were negative for *RUNX1-RUNX1T1* in the first 3 months after allo-HSCT (14). Therefore, with the help of preemptive IFN-α therapy, patients with low-level and intermediate-level *RUNX1-RUNX1T1* achieved comparable outcomes with those with persistent MRD-negative status after allo-HSCT. Patients with low-level and intermediate-level *RUNX1-RUNX1T1* have been suggested to benefit more from IFN-α therapy, which could preferably be started in patients with a relatively low tumor burden (48).

In patients with high-level *RUNX1-RUNX1T1*, neither preemptive DLI nor IFN-α showed satisfactory outcomes. The survival of the IFN-α and DLI groups was comparable due to the small sample size of the DLI recipients. Furthermore, patients with high-level *RUNX1-RUNX1T1* who showed an unsatisfactory response to DLI achieved MRD negative after salvage IFN-α treatment; however, those who showed an unsatisfactory response to IFN-α did not benefit from salvage DLI. We also observed that IFN-α salvage treatment was effective for patients who did not respond satisfactorily to preemptive DLI (18, 49). The number of patients who received both IFN-α and DLI was too small to draw any conclusions in this study, but this was an interesting phenomenon that suggested that therapeutic order may influence the outcomes of preemptive immunotherapy, and it is worth identifying in the future. Meanwhile, many new drugs (e.g., BCL-2 inhibitor) could be used in the treatment of AML, which would help to further improve the clinical outcomes of patients with high-level *RUNX1-RUNX1T1* (50–52).

This was not a randomized trial, which was a limitation of the present study. Many patients might be inclined to choose IFN-α therapy because it can be conveniently performed in an outpatient setting, particularly for those with low-level and intermediate-level *RUNX1-RUNX1T1*. Therefore, it was too early to draw strong conclusions that DLI was inferior to IFN-α therapy in these patients, which should be confirmed by a randomized trial. Secondly, the number of patients with high-level *RUNX1-RUNX1T1* was small. Because most of the patients with low-level and intermediate-level *RUNX1-RUNX1T1* could clear the MRD after IFN-α therapy, the evolution of MRD was stopped in the early stage and did not develop into high-level *RUNX1-RUNX1T1*. Thus, the efficacy of DLI and IFN-α therapy in patients with high level of *RUNX1-RUNX1T1* should also be further investigated.

## Conclusion

This study showed that patients with low-level and intermediate-level *RUNX1-RUNX1T1* could benefit more from preemptive IFN-α therapy compared with DLI. Clinical outcomes were comparable between preemptive IFN-α therapy and DLI in patients with high-level *RUNX1-RUNX1T1*; however, they should be further improved. In the future, randomized trials will compare the efficacy of IFN-α therapy with that of DLI in these patients.

## Data Availability Statement

The raw data supporting the conclusions of this article will be made available by the authors, without undue reservation.

## Author Contributions

X-DM and X-JH designed the study. M-ZS, SF, X-HZ, L-PX, YW, C-HY, HC, Y-HC, WH, F-RW, J-ZW, X-SZ, Y-ZQ, Y-JC, and K-YL collected the data. M-ZS, SF, X-DM, and X-JH analyzed the data and drafted the manuscript. All authors contributed to the data interpretation, manuscript preparation, and approval of the final version.

## Funding

This work was supported by the National Key Research and Development Program of China (grant number 2017YFA0104500), CAMS Innovation Fund for Medical Sciences (CIFMS) (grant number 2019-I2M-5-034), the Capital’s Funds for Health Improvement and Research (grant number 2018-4-4089), the Foundation for Innovative Research Groups of the National Natural Science Foundation of China (grant number 81621001), the Program of the National Natural Science Foundation of China (grant number 82170208), the Key Program of the National Natural Science Foundation of China (grant number 81930004), and the Fundamental Research Funds for the Central Universities.

## Conflict of Interest

The authors declare that the research was conducted in the absence of any commercial or financial relationships that could be construed as a potential conflict of interest.

## Publisher’s Note

All claims expressed in this article are solely those of the authors and do not necessarily represent those of their affiliated organizations, or those of the publisher, the editors and the reviewers. Any product that may be evaluated in this article, or claim that may be made by its manufacturer, is not guaranteed or endorsed by the publisher.
