# Supplementary material for: Preemptive Immunotherapy for Minimal Residual Disease in Patients With t(8;21) Acute Myeloid Leukemia After Allogeneic Hematopoietic Stem Cell Transplantation
Source: Front Oncol. 2022 Jan 6;11:773394. doi: 10.3389/fonc.2021.773394 (PMC8770808; doi:10.3389/fonc.2021.773394)
Supplement: Supplementary file 1 [file DataSheet_1.docx]

**Supplementary methods**

**Transplant regimens**

Preconditioning consisted of cytarabine (Ara-C), busulfan (3.2 mg·kg^−1^·day^−1^ administered intravenously on days −8 to −6) (day 0 being the first day of donor cell infusion), cyclophosphamide (CY, 1.8 g·m^−2^·day^−1^, days −5 to −4), and semustine (250 mg^.^m^−2^, day −3). Ara-C was administered at 4 g·m^−2^·day^−1^ (days −10 to −9) to the human leukocyte antigen (HLA)-haploidentical donor (HID) group, at 2 g·m^−2^·day^−1^ (days −10 to −9) to the HLA-unrelated donor (URD) group, and at 2 g·m^−2^·day^−1^ (day −9) to the HLA-identical sibling donor (ISD) group. Rabbit antithymocyte globulin (thymoglobulin, 2.5 mg·kg^−1^·day^−1^, days −5 to −2; Sanofi, France) was administered to the HID and URD groups. Particularly, the patients with mother donors or collateral relative donors received two doses of 14.5 mg/kg CY on days 3 and 4 post-HSCT according to the protocol of clinical trial registered at http://clinicaltrials.gov/NCT02412423. Granulocyte colony-stimulating factor (G-CSF)-mobilized, fresh, and unmanipulated bone marrow (BM) and/or peripheral blood harvests were infused into the recipients on the day of collection. In addition, patients received cyclosporine A (CSA), mycophenolate mofetil (MMF), and short-term methotrexate (MTX) as GVHD prophylaxis.

**Definition of CR**

CR designation requires that the patient achieve the morphologic leukemia-free state (Morphologic leukemia-free state: This designation requires less than 5% blasts in an aspirate sample with marrow spicules and with a count of at least 200 nucleated cells. There should be no blasts with Auer rods or persistence of extramedullary disease) and have an absolute neutrophil count of more than 1,000/μL and platelets of ≥100,000/μL. Hemoglobin concentration or hematocrit has no bearing on remission status, although the patient must be independent of transfusions.

**MRD monitoring and definition**

MRD was monitored as the level of *RUNX1-RUNX1T1* transcripts in patients with t(8;21) AML. The expressions of *RUNX1-RUNX1T1* were evaluated by TaqMan-based real time quantitative reverse transcription polymerase chain reaction (RQ-PCR). We selected ABL as a control gene. The experiments were performed in duplicate. The transcript level was calculated as *RUNX1-RUNX1T1* transcript copies/ABL copies as a percentage. The pretreatment baseline level of *RUNX1-RUNX1T1* transcripts was 388% in our laboratory, and *RUNX1-RUNX1T1* transcripts positivity was defined as a <4.5-log reduction in *RUNX1-RUNX1T1* transcripts when compared with the pretreatment baseline level and/or the loss of a ≥4.5-log reduction after 3 months post-HSCT. Routine MRD monitoring was performed 1, 2, 3, 4.5, 6, 9, and 12 months post-transplantation and at 6-month intervals thereafter.

**Preemptive intervention protocol**

Recombinant human IFN-α-2b injections (Anferon; Tianjin Hualida Biotechnology Co., Ltd., Tianjin, China) were administered subcutaneously twice weekly in every 4 weeks cycle at dosages of 3 million units for patients older than 16 years, and at 3 million units per square meter for those younger than 16 years (capped by 3 million units). IFN-α therapy was scheduled for 6 cycles or until *RUNX1-RUNX1T1* transcripts were negative at least for 2 consecutive tests, and prolonged IFN-α therapy was permitted at the request of patients. Adverse events were scored using the National Cancer Institute Common Toxicity Criteria version 4.0, and they were monitored every 1-2 weeks after IFN-α therapy. GVHD was excluded as an adverse event. Study medication with IFN-α was discontinued in any patient with active GVHD (grade II or higher aGVHD or cGVHD with moderate or higher severity), severe infection, grade ≥ 3 toxicity, relapse, or non-relapse mortality (NRM).

G-CSF–mobilized peripheral blood stem cells were administered instead of the more common unstimulated donor blood lymphocytes. Patients also received anti-leukemic chemotherapy 48–72 hours before DLI (i.e., Chemo-DLI). If the patients who did not want to receive chemotherapy, they could receive DLI alone, particularly for the patients with low- and intermediate-level *RUNX1-RUNX1T1*. Chemotherapy regimens for the initial Chemo-DLI included AA (aclacinomycin 10 mg∙m^−2^∙day^−1^ for 5 days and Ara-C 100 mg∙m^−2^∙day^−1^ for 5 days; n=4), Ara-C+VP16 (Ara-c 1.2 g∙m^−2^∙day^−1^ for 3 days and VP-16 60 mg∙m^−2^∙day^−1^ for 2 days; n=1), HA (harringtonine 2 mg∙m^−2^∙day^−1^ for 5 days and Ara-C 100 mg∙m^−2^∙day^−1^ for 5 days; n=2), HAA (harringtonine 2 mg∙m^−2^∙day^−1^ for 5 days, aclacinomycin 10 mg∙m^−2^∙day^−1^ for 5 days, and Ara-C 100 mg∙m^−2^∙day^−1^ for 5 days; n=2), FLAG (Fludarabine 50 mg∙m^−2^∙day^−1^ for 5 days; Ara-C 2.0 mg∙m^−2^∙day^−1^ for 5 days, and G-CSF 0.3 mg∙m^−2^∙day^−1^ for 6 days; n=1). Chemotherapy regimens for the salvage Chemo-DLI after IFN-α included AA (n=6), Ara-C+VP16 (n=1), HA (n=1), HAA (n=10), MA (mitoxantrone 6~8 mg∙m^−2^∙day^−1^ for 3 days and Ara-C 100 mg∙m^−2^∙day^−1^ for 7 days; n=1), IA (idarubicin 10 mg∙m^−2^∙day^−1^ for 2 days and Ara-C 500 mg∙m^−2^∙day^−1^ for 3 days; n=1). G-CSF–mobilized peripheral blood stem cells were administered instead of the unstimulated donor blood lymphocytes, and the median doses of mononuclear cells were 1.0 (1.0–6.7) × 10^8^/kg. Patients received immunosuppressive drugs (e.g., CSA or MTX) to prevent GVHD after DLI. Patients receiving DLI from an ISD received GVHD prophylaxis for 4–6 weeks, while those receiving DLI from a HID or URD received GVHD prophylaxis for 6–8 weeks at the discretion of the attending physicians (and usually depending on the patient’s GVHD status after DLI). The starting dosage of CSA was 2.5 mg·kg^−1^·day^−1^, which was adjusted to maintain a plasma concentration >100 ng/mL.

MRD status was monitored 1, 2, 3, 4.5, 6, 9, and 12 months after preemptive interventions and at 6-month intervals thereafter.

For patients who regained MRD positivity after achieving MRD-negative status, or those with persistent and increasing levels of MRD (e.g., 1-log rising levels of *RUNX1-RUNX1T1* transcripts), if they were in the IFN-α group, they were switched to DLI group; if they were in the DLI group, they were switched to IFN-α group.

**Treatment of GVHD after preemptive interventions**

Acute GVHD (aGVHD) was treated with methylprednisolone (1–2 mg∙kg^-1^ per day) and by resumption of full-dose CSA administration. Second- or third-line immunosuppressive therapies such as CD25 monoclonal antibody (Basiliximab; Novartis Pharma Stein AG, Basel, Switzerland), MMF, tacrolimus, or MTX were administered in cases of steroid-refractory aGVHD. Moderate to severe chronic GVHD (cGVHD) was treated with prednisone (1 mg∙kg^-1^ per day), and CSA was adjusted to maintain a trough blood concentration >150 ng/mL. Second- or third-line immunosuppressive therapies such as MMF, MTX, penicillamine, azathioprine, rituximab, or tacrolimus were administered in cases of steroid-refractory cGVHD.
