# Supplementary material for: Preemptive Immunotherapy for Minimal Residual Disease in Patients With t(8;21) Acute Myeloid Leukemia After Allogeneic Hematopoietic Stem Cell Transplantation
Source: Front Oncol. 2022 Jan 6;11:773394. doi: 10.3389/fonc.2021.773394 (PMC8770808; doi:10.3389/fonc.2021.773394)
Supplement: Supplementary file 3 [file Table_1.docx]

**Supplementary table 1. The characteristics of the patients with HLA-identical sibling donor and HLA-haploidentical related donor.**

| **Characteristics** | **IFN-α group**  **(*n*=85)** | |  | **DLI group**  **(*n*=16)** | |  | **HLA-haploidentical related donor (n=77)** | **HLA-identical sibling donor (n=24)** | ***P* value** |
| --- | --- | --- | --- | --- | --- | --- | --- | --- | --- |
|  | HLA-haploidentical related donor  （n=65） | HLA-identical sibling donor  （n=20） | ***P* value** | HLA-haploidentical related donor  （n=12） | HLA-identical sibling donor（n=4) | ***P* value** |  |  |  |
| Median age at allo-HSCT, years (range) | 27 (7-46) | 36 (8-57) | 0.008 | 30.5 (15-49) | 28.5 (4-34) | 0.770 | 27 (7-49) | 33 (4-57) | 0.023 |
| Median time from allo-HSCT to interventions, days (range) | 141 (36-556) | 132 (42-710) | 0.565 | 102.5 (46-217) | 125.5 (63-184) | 0.953 | 140 (36-556) | 132 (42-710) | 0.582 |
| First CR induction courses, *n* (%) |  |  | 0.031 |  |  | 1.000 |  |  | 0.060 |
| 1 | 54 (83.1) | 12 (60.0) |  | 6 (50.0) | 2 (50.0) |  | 60 (77.9) | 14 (58.3) | 0.937 |
| > 1 | 11 (16.9) | 8 (40.0) |  | 6 (50.0) | 2 (50.0) |  | 17 (22.0) | 10 (41.6) |  |
| *c-KIT* gene at diagnosis, *n* (%) |  |  | 0.717 |  |  | 0.684 |  |  | 0.937 |
| Mutation | 29 (44.6) | 8 (40.0) |  | 7 (58.3) | 3 (75.0) |  | 41 (53.2) | 11 (45.8) |  |
| Wild type | 36 (55.4) | 12 (60.0) |  | 5 (41.7) | 1 (25.0) |  | 36 (46.7) | 13 (54.1) |  |
| Sex, *n* (%) |  |  | 0.693 |  |  | 0.262 |  |  | 0.340 |
| Male | 39 (60.0) | 11 (55.0) |  | 8 (66.7) | 1 (25.0) |  | 47 (61.0) | 12 (50.0) |  |
| Female | 26 (40.0) | 9 (45.0) |  | 4 (33.3) | 3 (75.0) |  | 30 (38.9) | 12 (50.0) |  |
| Disease status at allo-HSCT, *n* (%) |  |  | 0.422 |  |  | 0.170 |  |  | 0.898 |
| CR1 | 54 (83.1) | 15 (75.0) |  | 6 (50.0) | 4 (100.0) |  | 60 (77.9) | 19 (79.1) |  |
| > CR1 | 11 (16.9) | 5 (25.0) |  | 6 (50.0) | 0 (0.0) |  | 17 (22.0) | 5 (20.8) |  |
| *RUNX1-RUNX1T1* transcript levels before HSCT, *n* (%) |  |  | 1.000 |  |  | 0.133 |  |  | 0.547 |
| 3.5-4.5-log reduction | 11 (16.9) | 2 (10.0) |  | 1 (8.3) | 0 (0.0) |  | 12 (15.5) | 2 (8.3) |  |
| 2.5-3.5-log reduction | 28 (43.1) | 11 (55.0) |  | 1 (8.3) | 3 (75.0) |  | 29 (37.6) | 14 (58.3) |  |
| <2.5-log reduction | 26 (40.0) | 7 (35.0) |  | 10 (83.3) | 1 (25.0) |  | 36 (46.7) | 8 (33.3) |  |
| Donor-recipient sex match, *n* (%) |  |  | 0.708 |  |  | 0.521 |  |  | 0.520 |
| Male-male | 28 (43.1) | 4 (20.0) |  | 5 (41.7) | 1 (25.0) |  | 33 (42.8) | 5 (20.8) |  |
| Male-female | 19 (29.2) | 7 (35.0) |  | 3 (25.0) | 1 (25.0) |  | 22 (28.5) | 8 (33.3) |  |
| Female-male | 10 (15.4) | 7 (35.0) |  | 3 (25.0) | 0 (0.0) |  | 13 (16.8) | 7 (29.1) |  |
| Female-female | 8 (12.3) | 2 (10.0) |  | 1 (8.3) | 2 (50.0) |  | 9 (11.6) | 4 (16.6) |  |
| *RUNX1-RUNX1T1* level before interventions, *n* (%) ^a^ |  |  | 0.184 |  |  | 0.599 |  |  | 0.119 |
| Low | 38 (58.5) | 9 (45.0) |  | 3 (25.0) | 0 (0.0) |  | 41 (53.2) | 9 (37.5) |  |
| Intermediate | 22 (33.8) | 7 (35.0) |  | 4 (33.3) | 2 (50.0) |  | 26 (33.7) | 9 (37.5) |  |
| High | 5 (7.7) | 4 (20.0) |  | 5 (41.7) | 2 (50.0) |  | 10 (12.9) | 6 (25.0) |  |
| GVHD prophylaxis protocol, *n* (%) |  |  |  |  |  |  |  |  |  |
| ATG | 60 (92.3) |  |  | 11 (91.6) |  |  | 71 (92.2) |  |  |
| ATG+low dose PTCY | 5 (7.7) |  |  | 1 (8.3) |  |  | 6 (7.8) |  |  |
| Discontinuing immunosuppressions before interventions, *n* (%) | 34 (52.3) | 13 (65.0) | 0.321 | 5 (41.7) | 3 (75.0) | 0.379 | 39 (50.6) | 16 (66.6) | 0.171 |
| aGVHD before MRD positive, *n* (%) | 31 (47.6) | 0 (0.0) | <0.001 | 7 (58.3) | 1 (25.0) | 0.379 | 38 (49.3) | 1 (4.1) | <0.001 |
| cGVHD before MRD positive, *n* (%) | 2 (3.0) | 1 (5.0) | 0.685 | 0 (0) | 0 (0) | 1.000 | 2 (2.6) | 1 (4.2) | 0.694 |
| allo-HSCT, allogeneic hematopoietic stem cell transplantation; CR, complete remission; DLI, donor lymphocyte infusion; GVHD, graft-versus-host disease; HLA, human leukocyte antigen; IFN-α, interferon-α; ATG, antithymocyte globulin; PTCY, post-transplant CY.  ^a^ High-level, intermediate-level, and low-level MRDs were respectively defined as <2.5-log, 2.5 to 3.5-log and 3.5 to 4.5-log reductions in the *RUNX1-RUNX1T1* transcripts when compared with the pretreatment baseline level. | | | | | | | | | |
