# Supplementary material for: Preemptive Immunotherapy for Minimal Residual Disease in Patients With t(8;21) Acute Myeloid Leukemia After Allogeneic Hematopoietic Stem Cell Transplantation
Source: Front Oncol. 2022 Jan 6;11:773394. doi: 10.3389/fonc.2021.773394 (PMC8770808; doi:10.3389/fonc.2021.773394)
Supplement: Supplementary file 4 [file Table_2.docx]

| **Characteristics** | ***RUNX1-RUNX1T1* transcript levels after HSCT** | |
| --- | --- | --- |
|  | **Correlation coefficient (*r*)** | ***p*** |
| *RUNX1-RUNX1T1* transcript levels before HSCT | 0.308 | <0.001 |
| Donor type | 0.131 | 0.185 |
| *c-KIT* gene at diagnosis | 0.145 | 0.142 |
| Other karyotypes | -0.081 | 0.414 |

**Supplementary table 2. Correlation analysis between patient characteristics and *RUNX1-RUNX1T1* transcript levels after HSCT**

HSCT, allogeneic hematopoietic stem cell transplantation;
