# Supplementary material for: Preemptive Immunotherapy for Minimal Residual Disease in Patients With t(8;21) Acute Myeloid Leukemia After Allogeneic Hematopoietic Stem Cell Transplantation
Source: Front Oncol. 2022 Jan 6;11:773394. doi: 10.3389/fonc.2021.773394 (PMC8770808; doi:10.3389/fonc.2021.773394)
Supplement: Supplementary file 5 [file Table_3.docx]

**Supplementary table 3. Median duration of immunosuppressive therapy before MRD occurred**

|  | **RUNX1-RUNX1T1 transcript levels after HSCT** | | |  |
| --- | --- | --- | --- | --- |
|  | High-level | Intermediate-level | Low-level | P value |
| Median duration of immunosuppressive therapy before MRD occurred, days (range) | 47 (27-199) | 60 (21-280) | 85 (21-324) | 0.816 |

HSCT, hematopoietic stem cell transplantation;

^a^ High-level, intermediate-level, and low-level MRDs were respectively defined as <2.5-log, 2.5 to 3.5-log and 3.5 to 4.5-log reductions in the *RUNX1-RUNX1T1* transcripts when compared with the pretreatment baseline level.
