# Supplementary material for: Preemptive Immunotherapy for Minimal Residual Disease in Patients With t(8;21) Acute Myeloid Leukemia After Allogeneic Hematopoietic Stem Cell Transplantation
Source: Front Oncol. 2022 Jan 6;11:773394. doi: 10.3389/fonc.2021.773394 (PMC8770808; doi:10.3389/fonc.2021.773394)
Supplement: Supplementary file 6 [file Table_4.docx]

**Supplementary table 4.** **The causes of NRM after preemptive interventions**

| **Causes of death** | IFN-α group (n=64) | DLI group (n=10) | IFN-α+DLI group (n=30) |
| --- | --- | --- | --- |
| Infection | 3 | 0 | 2 |
| DAH | 0 | 0 | 1 |
| GVHD | 0 | 1 | 0 |

DAH, diffuse alveolar hemorrhage; DLI, donor lymphocyte infusion; GVHD, graft-versus-host disease; IFN-α, interferon-α; NRM, non-relapse mortality.
