# Supplementary material for: Preemptive Immunotherapy for Minimal Residual Disease in Patients With t(8;21) Acute Myeloid Leukemia After Allogeneic Hematopoietic Stem Cell Transplantation
Source: Front Oncol. 2022 Jan 6;11:773394. doi: 10.3389/fonc.2021.773394 (PMC8770808; doi:10.3389/fonc.2021.773394)
Supplement: Supplementary file 7 [file Table_5.docx]

**Supplementary table 5. The rate of MRD achieving negative after interventions**

|  | **MRD achieving negative** | |
| --- | --- | --- |
| **Time after interventions, months** | **IFN-α group, *n* (%)** | **DLI group, *n* (%)** |
| Low-level *RUNX1-RUNX1T1* ^a^ | 48 | 3 |
| 1 | 20 (41.6) | 0 (0) |
| 2 | 6 (12.5) | 0 (0) |
| 3 | 6 (12.5) | 0 (0) |
| >3 | 10 (20.8) | 0 (0) |
| Persistent positive | 6 (12.5) | 3 (100) |
| Intermediate-level *RUNX1-RUNX1T1* ^a^ | 31 | 6 |
| 1 | 6 (19.3) | 1 (16.6) |
| 2 | 2 (6.4) | 0 (0) |
| 3 | 1 (3.2) | 0 (0) |
| >3 | 9 (29.0) | 2 (33.3) |
| Persistent positive | 13 (41.9) | 3 (50.0) |
| High-level *RUNX1-RUNX1T1* ^a^ | 9 | 7 |
| 1 | 1 (11.1) | 0 (0) |
| 2 | 1 (11.1) | 1 (14.3) |
| 3 | 0 (0) | 0 (0) |
| >3 | 0 (0) | 0 (0) |
| Persistent positive | 7 (77.8) | 6 (85.7) |

DLI, donor lymphocyte infusion; IFN, interferon; MRD, minimal residual disease.

^a^ High-level, intermediate-level, and low-level MRDs were respectively defined as <2.5-log, 2.5 to 3.5-log and 3.5 to 4.5-log reductions in the *RUNX1-RUNX1T1* transcripts when compared with the pretreatment baseline level.
