# Supplementary material for: Preemptive Immunotherapy for Minimal Residual Disease in Patients With t(8;21) Acute Myeloid Leukemia After Allogeneic Hematopoietic Stem Cell Transplantation
Source: Front Oncol. 2022 Jan 6;11:773394. doi: 10.3389/fonc.2021.773394 (PMC8770808; doi:10.3389/fonc.2021.773394)
Supplement: Supplementary file 8 [file Table_6.doc]

**Supplementary table 6. Multivariate analysis of risk factors for the 2-year clinical outcomes after IFN-α therapy in per protocol set**

| **Outcome** | **HR (95% CI)** | ***P*** |
| --- | --- | --- |
| **Relapse** |  |  |
| Disease status prior to allo-HSCT |  |  |
| CR1 | 1 |  |
| > CR1 | 4.13 (0.45-37.58) | 0.208 |
| MRD level before IFN-α treatment ^a^ |  |  |
| High-level | 1 |  |
| Intermediate-level | 0.02 (0.00-0.36) | 0.008 |
| Low-level | 0.05 (0.00-0.60) | 0.018 |
| Donor type |  |  |
| Alternative donor | 1 |  |
| HLA-identical donor | 46.57 (3.75-578.79) | 0.003 |
| **Treatment failure as defined by OS** |  |  |
| MRD level before IFN-α treatment ^a^ |  |  |
| High-level | 1 |  |
| Intermediate-level | 0.04 (0.00-0.40) | 0.006 |
| Low-level | 0.08 (0.01-0.58) | 0.012 |
| Donor type |  |  |
| Alternative donor | 1 |  |
| HLA-identical donor | 24.57 (3.94-153.33) | 0.001 |
| **Treatment failure as defined by LFS** |  |  |
| Disease status prior to allo-HSCT |  |  |
| CR1 | 1 |  |
| > CR1 | 3.13 (0.773-12.70) | 0.110 |
| Donor type |  |  |
| Alternative donor | 1 |  |
| HLA-identical donor | 18.39 (3.80-88.98) | <0.001 |

allo-HSCT, allogeneic hematopoietic stem cell transplantation; CI, confidence interval; HR, hazard ratio; HLA, human leukocyte antigen; IFN-α, interferon-α; LFS, leukemia-free survival; MRD, minimal residual disease; OS, overall survival.

^a^ High-level, intermediate-level, and low-level MRDs were respectively defined as <2.5-log, 2.5 to 3.5-log and 3.5 to 4.5-log reductions in the *RUNX1-RUNX1T1* transcripts.
