# Supplementary material for: Preemptive Immunotherapy for Minimal Residual Disease in Patients With t(8;21) Acute Myeloid Leukemia After Allogeneic Hematopoietic Stem Cell Transplantation
Source: Front Oncol. 2022 Jan 6;11:773394. doi: 10.3389/fonc.2021.773394 (PMC8770808; doi:10.3389/fonc.2021.773394)
Supplement: Supplementary file 9 [file Table_7.docx]

**Supplementary table 7. The 2-year incidence of relapse, NRM, LFS and OS after combined preemptive interventions in low- and intermediate-level *RUNX1-RUNX1T1* patients**

|  |  | IFN-α followed by DLI |  | DLI followed by IFN-α |  |
| --- | --- | --- | --- | --- | --- |
|  | n=21 | Cumulative incidence  (95%CI) | n=4 | Cumulative incidence  (95%CI) | *P* value |
| Relapse | 8 | 29.8% (9.2%-50.5%) | 0 | 0.0% | 0.209 |
| NRM | 0 | 0.0% | 2 | 50.0% (0.0%-100.0%) | 0.001 |
| LFS | 13 | 70.2% (52.8%-93.3%) | 2 | 50.0% (18.8%-100.0%) | 0.420 |
| OS | 16 | 80.0% (64.26%-99.6%) | 2 | 50.0% (18.8%-100.0%) | 0.120 |

CI, confidence interval; DLI, donor lymphocyte infusion; IFN-α, interferon-α; LFS, leukemia-free survival; NRM, non-relapse mortality; OS, overall survival.
